# Global Screening of *Salmonella enterica* Serovar Typhimurium Genes for Desiccation Survival

**DOI:** 10.3389/fmicb.2017.01723

**Published:** 2017-09-08

**Authors:** Rabindra K. Mandal, Young M. Kwon

**Affiliations:** ^1^Department of Poultry Science, University of Arkansas Fayetteville, AR, United States; ^2^Cell and Molecular Biology Program, University of Arkansas Fayetteville, AR, United States

**Keywords:** *Salmonella*, desiccation stress, genetic determinants, Tn-seq, low water activity food

## Abstract

*Salmonella* spp., one of the most common foodborne bacterial pathogens, has the ability to survive under desiccation conditions in foods and food processing facilities for years. This raises the concerns of *Salmonella* infection in humans associated with low water activity foods. *Salmonella* responds to desiccation stress via complex pathways involving immediate physiological actions as well as coordinated genetic responses. However, the exact mechanisms of *Salmonella* to resist desiccation stress remain to be fully elucidated. In this study, we screened a genome-saturating transposon (Tn5) library of *Salmonella* Typhimurium (*S*. Typhimurium) 14028s under the *in vitro* desiccation stress using transposon sequencing (Tn-seq). We identified 61 genes and 6 intergenic regions required to overcome desiccation stress. *Salmonella* desiccation resistance genes were mostly related to energy production and conversion; cell wall/membrane/envelope biogenesis; inorganic ion transport and metabolism; regulation of biological process; DNA metabolic process; ABC transporters; and two component system. More than 20% of the *Salmonella* desiccation resistance genes encode either putative or hypothetical proteins. Phenotypic evaluation of 12 single gene knockout mutants showed 3 mutants (*atpH, atpG*, and *corA*) had significantly (*p* < 0.02) reduced survival as compared to the wild type during desiccation survival. Thus, our study provided new insights into the molecular mechanisms utilized by *Salmonella* for survival against desiccation stress. The findings might be further exploited to develop effective control strategies against *Salmonella* contamination in low water activity foods and food processing facilities.

## Introduction

*Salmonella* is one of the most common causes of foodborne illness worldwide. It can withstand a spectrum of hostile milieus such as, desiccation found in natural and food industry settings (Spector and Kenyon, 2012). *Salmonella* can persist in the low water activity (low-a_w_) environment for extended periods of time. *Salmonella* is also able to survive from several weeks and months, even to years in dry foods (chocolate, hard cheese, dried eggs, infant dried milk, salami, halva, almonds kernels, pecans, dry confectionery raw materials, and peanut-flavored candy; Beuchat and Heaton, 1975; Kotzekidou, 1998; Hiramatsu et al., 2005; Uesugi et al., 2006; Bell and Kyriakides, 2008; Komitopoulou and Peñaloza, 2009; Gruzdev et al., 2012a) and dry surfaces (desiccated paper discs, plastics, and eggshells; Braun et al., 1999). Globally, there were 7,315 reported cases of bacterial outbreak illness and 63 deaths due to consumption of contaminated low-a_w_ foods and spices during the period from 2007 to 2012. *Salmonella* alone was accountable for 94% of the low-a_w_ food recalls in the U.S. and 53% of outbreaks worldwide in the above 6 years (Farakos and Frank, 2014).

Additionally, exposure of *Salmonella* to low water activity increases cross-protection against other stresses including heat, ethanol, sodium hypochlorite, dodecyl dimethyl ammonium chloride, hydrogen peroxide, NaCl, bile salts, and UV irradiation (Gruzdev et al., 2011), which ultimately makes the prevention and control strategies less effective. Food industry faces a significant challenge to rein *Salmonella* burden from dry foods and spices without damaging the organoleptic properties. Control of *Salmonella* contamination in the low-a_w_ foods might be improved marginally through improvement in the hygiene and rapid and sensitive detection of *Salmonella* in food and food processing environments. However, it is more critical to understand the genetic mechanisms of *Salmonella* resistance in low-a_w_ environment for improvement of public food safety associated with low-a_w_ foods (Beuchat et al., 2013).

In the last few years, considerable progress has been made to unveil the underlying mechanisms of *Salmonella* tolerance against desiccation using transcriptome analysis (Deng et al., 2012; Gruzdev et al., 2012b; Li et al., 2012; Finn et al., 2013b). The immediate response of bacteria to low low-a_w_ foods environment involves balancing the internal osmotic pressure to keep them viable. Commonly believed mechanism for desiccation tolerance in *Salmonella* include the followings: increased potassium influx by *kdp* transporter; increased expression of osmoprotectant transport (*proPU* and *osmU*), glutamate and trehalose synthesis; and up-regulation of fatty acid catabolism, Fe-S cluster, sigma factors (*rpoE* and *rpoS*), and *ompC*. Additionally, cellulose and curli fimbriae may play an important role in desiccation resistance in *Salmonella*. However, Finn et al. (2015) reported that *S*. Typhimurium genes differentially expressed in response to different humectants, agents that reduce water content of food products, do not simply reflect low low-a_w_ but rather are linked to specific humectants (Finn et al., 2015). In addition, differentially expressed transcripts in a cell do not necessarily reflect the functional role of the genes at the given condition. Instead, the presence of these transcripts can be a reflection of the predictive adaptation of bacteria where the expressed transcripts may not have any functional roles in their current milieu (Tagkopoulos et al., 2008; Mitchell et al., 2009). Furthermore, the expression-based analysis does not provide insight into genes that are constitutively expressed or gene for which expression of the encoding proteins is controlled by post-transcriptional modifications. However, these limitations of expression-based analysis can be largely overcome by a more direct functional screening approach such as, transposon sequencing (Tn-seq) of saturated mutant libraries employed in this study.

In this study, for the first time to our knowledge, we used a Tn-seq approach to investigate the genetic determinants required for desiccation survival in *S*. Typhimurium. We screened a genome-saturating Tn5 mutant library of *S*. Typhimurium 14028s and identified 61 fitness genes required for survival of *S*. Typhimurium under a desiccation stress.

## Materials and methods

### Bacterial strains and growth conditions

*Salmonella enterica* serovar Typhimurium 14028S, a spontaneous mutant resistant to nalidixic acid (NA), was used for the transposon insertional mutagenesis. Bacteria were grown in Luria-Bertani (LB) medium or LB agar plates at 37°C and stored at −80°C unless indicated otherwise. NA (ICN Biomedicals Inc., Aurora OH, USA) and Kanamycin (Km, Shelton Scientific, Inc. CT, USA) were used at 25 μg/ml and 50 μg/ml, respectively. Bacteria were incubated on shaking rack at 225 rpm when required. Polystyrene disposable petri dishes (60 × 15 mm; VWR International, USA) were used for screening of the mutant library under a desiccation stress.

### Construction of Tn5 mutant library

Electrocompetent *S*. Typhimurium cells were prepared and transformed with EZ-Tn5 < KAN-2> Tnp transposome complex (Epicenter BioTechnologies, Madison, WI, USA) following the manufacturers' instructions. Electroporation was performed using 0.1-cm cuvettes in a Micropulser electroporator (Bio-Rad Laboratories, Inc., Mississauga, Ontario, Canada) with a field strength of 2450 V. The electroporated cells were immediately resuspended in 500 μl of SOC medium (Quality Biological Inc., Gaithersburg, MD) and incubated for 1.5 h at 37°C on a shaking rack (225 rpm). Then, the Tn5 mutant cells were plated on LB plates supplemented with double antibiotics (NA and Km), which were then incubated overnight at 37°C. We collected and combined ~370,000 Tn5 mutants from three transformations, making it a highly complex library. The mutant cells were scrapped off LB plates in 1X phosphate buffered saline (PBS; pH 7.0) and stored in 50% glycerol at −80°C.

### Screening of Tn5 mutant library during desiccation stress

The Tn5 complex library stored at −80°C was thawed on ice and 300 μl of the library was diluted in 60 ml LB and incubated at 37°C on a shaking rack for 30 min (OD_600_ = 0.135). Then, bacteria were collected by centrifugation at 5,500 rpm for 8 min at room temperature (RT) and resuspended in 50 ml PBS (OD_600_ = 0.143). Ten milliliter from this mutant suspension (*t*_0_ time point) were centrifuged and the bacterial pellet was saved for DNA extraction (Input pool; IP).

For negative selection of Tn5 mutants during desiccation survival, 10 ml of suspension from *t*_0_ was centrifuged and resuspended in one ml PBS (~8.0 × 10^8^ CFU/ml). Then aliquots of 100 μl were placed at the center of 10 petri plates (60 × 15 mm size) and air-dried with the lid open inside a biosafety hood with the blower on for 4 h. Then, the plates were covered with the lids and incubated at RT for 24 h. The desiccated cells were collected from all the 10 petri plates by resuspending them in one ml PBS buffer for each petri plate (in total 10 ml PBS), which was then concentrated in one ml PBS. Bacterial cells (100 μl aliquot) were plated on 10 LB plates (NA and Km) and incubated overnight. The cells were collected from all 10 plates in PBS, centrifuged and the pellet was stored at −20°C (Output pool; OP).

### DNA library preparation for illumina sequencing

Genomic DNA (gDNA) was extracted from IP and OP (100 μl aliquot of each pellet) using QIAamp DNA Mini Kit (Qiagen, Valencia, CA, USA) following manufacturer's protocol. The DNA was quantified using a Qubit 2.0 Fluorometer (Life Technologies, Carlsbad, CA). DNA libraries were prepared following the protocol developed in our lab (Dawoud et al., 2014) with some modifications. For detailed information, see Supplementary Protocol 1. Briefly, linear extension PCR was done to enrich the Tn5-juction sequences using a single primer specific to Tn5 transposon (7 bp upstream of invert repeat 2, IR2). The linear extension products were purified and C-tail was attached using terminal transferase (New England BioLabs, Ipswich, MA). C-tailed products were purified and exponential PCR was performed using barcoded forward primer and poly-G primer with an attached Illumina adapter (HTM primer; Table S1). The PCR products were separated on 1.5% agarose gel and the amplicons ranging from 300 to 500 bp were gel-purified. The purified DNA from IP and OP were mixed in an equal quantity (10 ng), and sent for Illumina sequencing using HiSeq 2000 single end read option with 100 cycles (Center for Genome Research and Biocomputing, Oregon State University, Corvallis).

### Data analysis

Sequencing reads obtained from the Illumina HiSeq 2000 single end read were analyzed using Analysis of high-Resolution Transposon-Insertion Sequences Technique (ARTIST; Pritchard et al., 2014). Briefly, demultiplexed reads with 20 bp transposon junction sequence were aligned against *S*. Typhimurium 14028s complete genome (accession number: NC_016856.1) using Bowtie version 0.12.7 (Langmead et al., 2009). The sequence alignment map (SAM) file was fed to ARTIST pipeline to identify conditionally essential genes (CEGs) using Con-ARTIST (Pritchard et al., 2014). Tn5 insertion reads were assigned to 100 bp windows of *S*. Typhimurium genome. Uncorrected raw data were used to normalize IP and then reads were compared between IP and OP using Mann-Whitney U test (MWU). The MWU results were used to train hidden Markov model (HMM) to predict the likelihood of loci to be conditionally essential or non-essential in OP (*p* < 0.01). Only the insertions in the middle 80% (excluding 10% at both 5′ and 3′ ends) of the protein-coding genes were considered to inactivate the protein functions and thus included in the analysis with cutoff >8 fold and >2 fold for depleted and enriched loci, respectively.

### Phenotypic evaluation of single gene knockout mutants

Single-gene knockout mutants of *S*. Typhimurium 14028s were ordered from the BEI Resources (www.beiresources.org; Porwollik et al., 2014). The mutants from 96-well plates were streaked on LB plates (Km) and grown overnight at 37°C. A single colony was picked and grown in LB broth (Km) for each mutant, and the strains were stored at −80°C in 50% glycerol. Twelve mutants were chosen based on the availability in our strain collection to represent the wide range of fold reduction in read numbers after the selection. Desiccation experiment was performed as described by Gruzdev et al. (2011) with some modifications (Gruzdev et al., 2011). A single colony of each mutant strain was picked from a LB plate with an appropriate antibiotic (NA for the wild type; Km for knockout mutants) and incubated in 10 ml LB broth with appropriate antibiotics aerobically overnight in standard conditions (37°C and shaking rack @225 rpm). Overnight grown bacteria were washed 3 times in 1X PBS at 4°C with centrifugation at 8,000 rpm for 2 min. O.D_600_ was adjusted to 0.4 (±0.05) in 1X PBS. Colony forming units (CFUs) were measured for the wild type and mutants. Fifty microliter of bacteria with adjusted OD_600_ (0.4) was then transferred to 96-microtiter well plates. The microtiter plate (with lid open) was placed inside a laminar flow hood with the blower on for 10 h to remove moisture. The bottom of the microtiter well was completely opaque after drying. After 10 h of drying the microtiter plate was covered with a lid and placed on the bench for additional 14 h at RT. Then 200 μl of 1X PBS was added to each well and the plate was shaken for 30 min at RT. The desiccated bacteria were then released from the wells and resuspended in PBS by vigorously pipetting 15 times and collected in 1.5 ml microcentrifuge tubes. The CFUs of the recovered viable cells was measured by plating the suspension on LB plates, followed by overnight incubation for each of the wild type and mutants. Three replications were performed for each strain. Survival (%) for each strain was calculated as (Total CFUs recovered after desiccation/ Total CFUs added) ^*^ 100.

## Results and discussion

### Overview of the selection process and illumina sequencing

**Figure 1:**
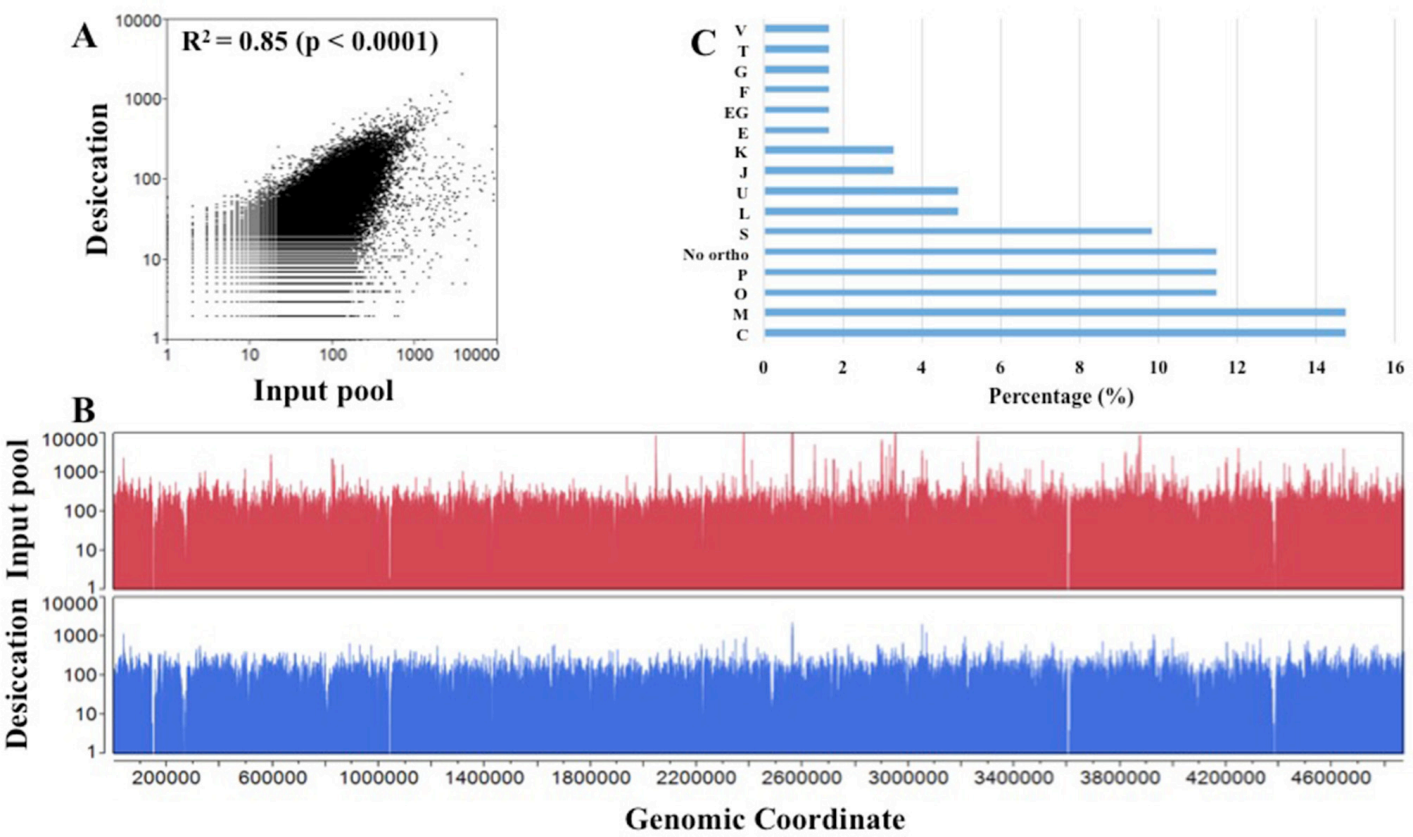
Overview of transposon sequencing. **(A)** Spearman correlation (*R*^2^) of Tn5 insertion raw reads frequency distribution between input pool and desiccation at the nucleotide level. X- and Y-axis are log transformed. **(B)** Overlay plot displays genome-wide Tn5 insertion distribution in input pool and desiccation at the nucleotide level (Table S1). **(C)** Cluster of orthologous group (COG) assigned to *S*. Typhimurium desiccation resistance genes using EggNOG 4.5 database. X-axis: Percentage of genes into each COG category and Y-axis: COG assignment. (C, Energy production and conversion; M, Cell wall/membrane/envelope biogenesis; O, Post-translational modification, protein turnover, and chaperones; P, Inorganic ion transport and metabolism; No ortho, No orthologous found; S, Function unknown; L, Replication, recombination and repair; U, Intracellular trafficking, secretion, and vesicular transport; J, Translation, ribosomal structure, and biogenesis; K, Transcription; E, Amino acid transport and metabolism; EG, Amino acid transport and metabolism, Carbohydrate transport and metabolism; F, Nucleotide transport and metabolism; G, Carbohydrate transport and metabolism; T, Signal transduction mechanisms; and V- Defense mechanisms).

In this experiment, we subjected a complex Tn5 library of *S*. Typhimurium with more than 350,000 mutants to a desiccation stress. The Tn5 library (IP) was air-dried for 4 h on petri plates inside a laminar flow hood and incubated at the room temperature for 24 h. The number of Tn5 mutants before and after the desiccation selection were 8 × 10^8^ CFU/ml and 8.7 × 10^6^ CFU/ml, respectively, indicating only 1.09% of the mutants were able to survive following the desiccation stress. This low recovery indicates the fact that desiccation is a harsh stress for survival of *Salmonella*. The desiccated Tn5 mutants were resuscitated on LB agar plates (Na and Km) under the standard growth condition. DNA was extracted from IP and OP and the Tn-seq amplicon libraries were prepared for HiSeq Illumina sequencing as described in Material and Methods. Illumina sequencing reads were demultiplexed based on a perfect match to sample barcodes and Tn5-junction sequences (20 bp) were extracted allowing some mismatches to the mosaic end of Tn5 (see Table S1). IP and OP had 10,842,764 and 5,516,907 reads, respectively, and more than 186,000 and 132,000 unique insertions, respectively (Table 1). The number of unique genomic sites (186,621) disrupted by Tn5 transposon in IP was unexpectedly lower than the number mutants collected (350,000) after electroporation of the transposome complex. This might be due to replication of Tn5 mutants during 1.5 h of incubation in SOC medium for phenotypic expression immediately after electroporation. Moderate Spearman's correlation was observed between IP and OP (*R*^2^ = 0.85, *p* < 0.0001) with Tn5 insertion frequency at the nucleotide level (Figure 1A; Table S2). Additionally, Tn5 transposons were randomly inserted throughout the entire genome without any noticeable genomic hot spots and amplification bias (Figure 1B). This reflects the good quality of the Tn5 mutant libraries (IP and OP) used for Illumina sequencing.

**Table 1:** Summary of Illumina sequencing reads.

**Library**	**Total reads**	**Reads mapped**	**Unique insertions (UnqIns)**	**Mean reads/UnqIns (±SE)**	**Median reads**
Input pool	10,842,764	8,867,116	186,621	48.99 ± 0.99	20
(IP)		(81%)			
Desiccation	5,516,907	4,248,156	132,631	33.18 ± 0.13	18
(OP)		(77%)			

### Identification of desiccation resistance genes

We used Con-ARTIST pipeline to identify the resistance genes required for the desiccation stress tolerance in *S*. Typhimurium. Con-ARTIST identifies transposon mutants at the single-insertion level and normalizes bottleneck effect enabling discovery of conditionally essential mutants at subgenic level (Pritchard et al., 2014). We identified 37 entirely conditionally essential (genes that contain significantly lower Tn5 reads throughout the entire coding regions) and 24 domain essential genes (genes that contain significantly lower Tn5 reads only in the certain region(s) of the entire coding sequences) that were required for survival during desiccation stress (Table 2). Among them, 10 genes encode putative proteins and six genes hypothetical proteins.

**Table 2:** The protein coding genes of *S*. Typhimurium 14028S required for desiccation survival.

**Locus_Tag (gene)**	**Protein annotation**	**COG symbol**	**Ess [log_2_FC] {DUIC}**
STM14_4660 (*atpC*)^n^^,^ ^s^^,^ ^o^	F0F1 ATP synthase subunit epsilon	C	1 [−5.16] {−10}
STM14_4661 (*atpD*)^n^^,^ ^s^^,^ ^o^	F0F1 ATP synthase subunit beta	C	2 [−3.58] {−39}
STM14_4662 (*atpG*)^n^^,^ ^s^^,^ ^o^	F0F1 ATP synthase subunit gamma	C	2 [−6.51] {−21}
STM14_4663 (*atpA*)^n^^,^ ^s^^,^ ^o^	F0F1 ATP synthase subunit alpha	C	2 [−7.25] {−27}
STM14_4664 (*atpH*)^n^^,^ ^s^^,^ ^o^	F0F1 ATP synthase subunit delta	C	2 [−5.97] {−10}
STM14_4665 (*atpF*)^n^^,^ ^s^^,^ ^o^	F0F1 ATP synthase subunit B	C	2 [−4.32] {−5}
STM14_4666 (*atpE*)^n^^,^ ^s^^,^ ^o^	F0F1 ATP synthase subunit C	C	2 [−5.11] {−13}
STM14_4667 (*atpB*)^n^^,^ ^s^^,^ ^o^	F0F1 ATP synthase subunit A	C	2 [−7.01] {−25}
STM14_4668 (*atpI*)^n^^,^ ^s^^,^ ^o^	F0F1 ATP synthase subunit I	C	2 [−0.44] {−4}
STM14_4723 (*wecE*)^s^	TDP-4-oxo-6-deoxy-D-glucose transaminase	E	2 [−2.94] {−9}
STM14_2256 (*pagO*)^s^	integral membrane protein	EG	2 [−2.16] {−23}
STM14_3075 (*guaA*)	bifunctional GMP synthase/glutamine amidotransferase protein	F	2 [−4.74] {−17}
STM14_4906 (*tpiA*)	triosephosphate isomerase	G	1 [−5.39] {−10}
STM14_3964 (*pnp*)	polynucleotide phosphorylase/polyadenylase	J	2 [−2.68] {−32}
STM14_5241 (*miaA*)	tRNA delta(2)-isopentenylpyrophosphate transferase	J	1 [−5.64] {−22}
STM14_4008 (*rpoN*)^o^	RNA polymerase factor sigma-54	K	1 [−5.08] {−18}
STM14_4722 (*wecD*)^s^	TDP-fucosamine acetyltransferase	K	1 [−5.43] {−8}
STM14_3676 (*xerD*)^o^	site-specific tyrosine recombinase XerD	L	2 [−3.7] {−11}
STM14_4196 (*dam*)^s^^,^ ^o^	DNA adenine methylase	L	1 [−4.2] {−20}
STM14_4750 (*xerC*)^s^^,^ ^o^	site-specific tyrosine recombinase XerC	L	2 [−7.81] {−8}
STM14_0265 (*yaeL*)^s^	zinc metallopeptidase	M	1 [−7.81] {−18}
STM14_0838	putative UDP-galactopyranose mutase	M	2 [−3.78] {−31}
STM14_0839	putative glycosyl transferase	M	1 [−1.93] {−12}
STM14_0871 (*pal*)^s^^,^ ^o^	peptidoglycan-associated outer membrane lipoprotein	M	2 [−3.8] {−4}
STM14_2580 (*rfbU*)^s^	mannosyl transferase	M	2 [−6.95] {−40}
STM14_2589 (*rfbA*)^s^	dTDP-glucose pyrophosphorylase	M	1 [−6.81] {−49}
STM14_3163 (*lepA*)^s^	GTP-binding protein LepA	M	2 [−2.33] {−17}
STM14_4656 (*glmS*)^o^	D-fructose-6-phosphate amidotransferase	M	2 [−5.37] {−26}
STM14_4724 (*wzxE*)^s^	O-antigen translocase	M	2 [−6.17] {−38}
STM14_0013 (*dnaK*)	molecular chaperone DnaK	O	2 [−5.39] {−12}
STM14_0014 (*dnaJ*)	chaperone protein DnaJ	O	1 [−1.26] {−17}
STM14_0254 (*glnD*)^o^	PII uridylyl-transferase	O	2 [−3.96] {−30}
STM14_2258^s^	putative inner membrane protein	O	1 [−1.15] {−23}
STM14_3328	putative inner membrane protein	O	2 [−2.11] {−25}
STM14_3675 (*dsbC*)^o^	thiol:disulfide interchange protein DsbC	O	1 [−0.13] {−4}
STM14_0048 (*nhaA*)^s^	pH-dependent sodium/proton antiporter	P	2 [−7.98] {−15}
STM14_0688 (*fepC*)	iron-enterobactin transporter ATP-binding protein	P	1 [−4.21] {−10}
STM14_0689 (*fepG*)	iron-enterobactin transporter permease	P	2 [−4.67] {−5}
STM14_0690 (*fepD*)	iron-enterobactin transporter membrane protein	P	2 [−4.46] {−4}
STM14_4648 (*phoU*)	transcriptional regulator PhoU	P	2 [−3] {−8}
STM14_4649 (*pstB*)^s^	phosphate transporter subunit	P	1 [−2.9] {−17}
STM14_4754 (*corA*)^o^	magnesium/nickel/cobalt transporter CorA	P	1 [−4.94] {−25}
STM14_0845	putative glycosyl transferase	S	1 [−0.41] {−18}
STM14_0872 (*ybgF*)^o^	hypothetical protein STM14_0872	S	1 [0.64] {−4}
STM14_1486	putative cytoplasmic protein	S	2 [−3.08] {−20}
STM14_3164 (*gogB*)^s^	hypothetical protein STM14_3164	S	2 [−0.63] {−34}
STM14_3329	putative inner membrane protein	S	1 [−2.64] {−8}
STM14_4907 (*yiiQ*)	hypothetical protein STM14_4907	S	1 [0.77] {−3}
STM14_5242 (*hfq*)^s^	RNA-binding protein Hfq	T	1 [−0.57] {−6}
STM14_0870 (*tolB*)^s^^,^ ^o^	translocation protein TolB	U	1 [−3.35] {−15}
STM14_1703 (*ssaH*)^s^	type III secretion system apparatus protein	U	2 [−0.58] {−1}
STM14_1705 (*ssaJ*)^s^	needle complex inner membrane lipoprotein	U	2 [−3.47] {−10}
STM14_5122	putative ABC-type bacteriocin/lantibiotic exporter	V	1 [−0.6] {−86}
STM14_1487	hypothetical protein STM14_1487	No ortho	2 [−1.59] {−3}
STM14_1490 (*envF*)	putative envelope lipoprotein	No ortho	2 [−0.58] {−7}
STM14_1704 (*ssaI*)^s^	type III secretion system apparatus protein	No ortho	2 [−5.47] {−10}
STM14_2015	hypothetical protein STM14_2015	No ortho	2 [−0.55] {−8}
STM14_3165	hypothetical protein STM14_3165	No ortho	2 [NA] {NA}
STM14_4725^s^	4-alpha-L-fucosyltransferase	No ortho	2 [−2.36] {−23}
STM14_5120	cation efflux pump	No ortho	1 [−1.56] {−23}

Ess, Essentiality based on Con-ARTIST; 1- Domain essential; 2, Entirely Essential; COG, Cluster of Orthologous Groups; log_2_FC, log2 fold change after read normalization in central 80% of gene; DUIC, difference of unique insertion count between input pool and desiccation in central 80% of gene; the more −ve more reduced is the fitness. COG annotations are similar as in Figure 1.

n, s, o *: Genes conditionally essential for in vitro osmotic (n), starvation (s) and oxidative stress (o) as shown in Figure 3 (Mandal, 2016) are indicated by the respective superscripts*.

Further, we assigned desiccation resistance gene to the cluster of orthologous groups (COG) using EggNOG 4.5 (http://eggnogdb.embl.de/#/app/home) with target taxa *Salmonella* (Table 2, Figure 1C). The desiccation resistance genes having no orthologous were assigned to “No orthologous group.” Equally highly abundant COGs were energy production and conversion (C), Cell wall/membrane/envelope biogenesis (M) (14.45%), followed by post-translational modification, protein turnover, and chaperones (O), and inorganic ion transport and metabolism (P) (both 11.48%). Additionally, the desiccation resistance genes belonging to no orthologous group and function unknown were also relatively higher (11.48 and 9.84%, respectively). The moderately abundant COGs were replication, recombination and repair (L), intracellular trafficking, secretion, and vesicular transport (U), translation, ribosomal structure and biogenesis (J) and transcription (K) ranging from 4.92 to 3.28%. Furthermore, COGs with only one gene were amino acid transport and metabolism (E), carbohydrate metabolism and transport (G), nucleotide transport and metabolism (F), signal transduction mechanisms (T), and defense mechanism (V). *pagO* belonged to both amino acid and carbohydrate metabolism and transport (Table 2; Figure 1C).

Additionally, we performed gene enrichment analysis using STRING database. The KEGG pathways and gene ontology (GO) process enriched for desiccation stress survival were searched in *S. enterica* LT2 (http://bit.ly/2cBK2e6). Genes that do not have orthologous genes in *S. enterica* LT2 background (STM14_1487, STM14_3165, and STM14_4725) were not considered. The abundant enriched categories included oxidative phosphorylation (ATP synthase genes), ABC transporters (*fepCDG, siiF*, and *pstB*), two component system (*glnD, rpoN*, and *pagO*), regulation of biological process (*hfq, rpoN, lepA, dsbC, dam*, and *glnD*), DNA metabolic process (*dam, dnaJK*, and *xerCD*) and O antigen biosynthetic process (*rfbU*, and *rfbA*).

### ATP synthase

All genes encoding the 9 subunits of ATP synthase were shown to be important for desiccation survival of *S*. Typhimurium (Table 2). ATP synthase is a highly conserved enzyme across the kingdoms of life with a pivotal role in chemiosmotic energy conversion. Bacteria when exposed to a desiccation stress, also suffer osmotic stress. Nouri and Komatsu (2010) found that during an osmotic stress in the soybean plant, H^+^-ATPases were the most prominent upregulated proteins, which help the plant maintain membrane potential for energy production, cell turgidity and intracellular pH (Nouri and Komatsu, 2010). Also, ATP synthase was one of the dominant proteins expressed over dehydration stress in chickpeas (Jaiswal et al., 2014). Additionally, in *Plectus murrayi*, bacteria feeding nematode, ATP synthase subunit transcripts were among the abundantly expressed under a desiccated condition (Adhikari et al., 2009).

### Cell wall/membrane/envelope biogenesis

The genes involved in cell wall/membrane/envelope biogenesis required by *S*. Typhimurium for desiccation survival were *rfbAU, wzxE, yaeL, pal, lepA, glmS*, STM14_0838, and STM14_0839. *rfbAU* are essential for O antigen (O polysaccharide) biosynthetic process. Polysaccharides in bacteria may act as a water reservoir in dry terrestrial environments. Garmiri et al. (2008) found that *Salmonella* spp. lacking O antigen are more sensitive to desiccation (Garmiri et al., 2008). Additionally, *wzxE* is involved in translocation of O antigen. STM14_0838 (putative UDP-galactopyranose mutase) encodes UDP-alpha-D-galactofuranose required for synthesis of cell wall in bacteria, fungi, and protozoa (http://www.genome.jp/dbget-bin/www_bget?ec:5.4.99.9). Furthermore, *Escherichia* coli *yaeL*, which encodes a membrane-bound zinc metalloprotease involved in regulated intramembrane proteolysis, is required for activation of sigma factor E (σ^E^) encoded by *rpoE* gene in response to an envelope stress (Kanehara et al., 2002). Mutation in *pal* gene (peptidoglycan associated lipoprotein) causes a severe defect in the cell envelope of gram-negative bacteria (Vines et al., 2005). *LepA*, ribosomal elongation factor 4 (EF4), has two opposing functions in *E. coli*—promoting survival during moderate stress by allowing stress-paused translation to resume and death during severe stress through self-destruction (Li et al., 2014).

### Post-translational modification, protein turnover, and chaperones

*Salmonella* desiccation resistance genes belonging to COG “O” category were *dnaJK, dsbC, glnD*, STM14_3328, STM14_2014, and STM14_2258. DnaK/DnaJ chaperone machinery is required for protein folding and essential for protein repair under both physiological and stressful conditions including heat shock stress (Takaya et al., 2004; Rychlik and Barrow, 2005). *E. coli* DsbC, a protein required for disulfide bond isomerization in periplasm, assists in folding of several envelope proteins containing disulfides formed between cysteine residues and is involved in the defense mechanism against oxidative stress (Denoncin et al., 2014). *E. coli* GlnD, a bifunctional uridylyltransferase/uridylyl-removing signal-transduction enzyme and the primary sensor of nitrogen status in cell, has a critical role in growth response to either nitrogen limitation or excess. Commonly, nitrogen is an essential chemical for all living being, which is an irreplaceable constituent of protein, DNA, and RNA (Tondervik et al., 2006; Yurgel et al., 2013). Probably, *S*. Typhimurium faces nitrogen limitation stress during desiccation in PBS, requiring *glnD* for survival. STM14_2258 (STM1864), a putative inner membrane protein, is regulated by RcsCDB system, which responds to envelope stress (Mariscotti and Garcia-del Portillo, 2009).

### Inorganic ion transport and metabolism

The *Salmonella* genes involved in inorganic ion transport and metabolism that are required for desiccation stress survival were *fepCDG, pstB, corA, nhaA*, and *phoU*. The *fepCDG* and *pstB* encode ATP-binding cassette (ABC) transporters. In *Rhizobium leguminosaurm*, a soil bacterium with the ability to fix nitrogen, mutation in an uncharacterized ABC transporter operon (RL2975–RL2977) caused the cell to be highly sensitive to desiccation stress due to significantly lower accumulation of exopolysaccharide (Vanderlinde et al., 2010). FepCDG are iron-enterobactin transporter, a high affinity siderophore that acquires iron for microbial systems (Porcheron et al., 2013). Virtually, iron is a vital nutrient for all forms of life and is required for energy generation, DNA replication, oxygen transport, and protection against oxidative stress (Skaar, 2010). Finn et al. (2013b) showed a number of genes involved in Fe-S clusters formation were upregulated during desiccation on a stainless steel surface which were induced under iron-limiting conditions (Finn et al., 2013b). Most bacteria regulate the uptake of inorganic orthophosphate (P_i_) by a negative regulatory protein PhoU via ABC phosphate-specific transporter (Pst). Phosphorous is an essential element in all cells with roles in diverse biological functions ranging from structural and metabolic biological processes to the composition of nucleic acids, phospholipids, and energy intermediates. However, we found only *pstB* (encoding cytoplasmic ATPase) and *phoU* were required for survival during desiccation in *S*. Typhimurium. The other three genes involved in P_i_ uptake systems includes extracellular P_i_ binding proteins (*pstS*) and two transmembrane channel proteins (*pstCA*) (Zheng et al., 2016). This might indicate the possibility of other redundant pathways doing the job of these three genes. Nonetheless, phosphate transport genes (*pstACS*) were differentially upregulated for the survival of desiccated *S*. Typhimurium on s stainless steel surface (Finn et al., 2013b).

CorA is a magnesium/nickel/cobalt transporter. The *corA* mutant of *Salmonella* shows a range of phenotypes including altered expression of *Salmonella* pathogenicity island 1(SPI-1) genes; decreased tolerance to heat shock and peroxide; defective invasion, survival, and proliferation inside macrophage and epithelial cells; decreased virulence and decreased tolerance to lactoperoxidase enzyme (Sermon et al., 2005). NhaA is pH-dependent sodium/proton (H^+^) antiporter that plays a critical role in intracellular pH regulation under alkaline conditions, cell volume regulation, and maintenance of electrochemical potential of Na^+^ across cytoplasmic membrane plus other (Vimont and Berche, 2000).

### Transcription (K) and replication, recombination and repair (L)

*Salmonella* desiccation tolerance gene related to transcription (K) were *rpoN* and *wecD*; and replication, recombination, and repair (L) were *dam* and *xerCD*. Alternative sigma factor 54 (σ^54^: encoded by *rpoN*) plays an important role in the regulation of stress resistance in many bacterial species. *E. coli* RpoN controls more than 14 operons/regulators during nitrogen-limiting conditions and protects the cells from alkaline pH during stationary-phase growth (Model et al., 1997; Reitzer and Schneider, 2001). Deletion of *rpoN* in *Listeria monocytogenes* affects the ability to grow under osmotic stress (Okada et al., 2006). Importantly, *Salmonella* Typhi RpoN regulates the expression of O-antigen, a water reservoir, during nitrogen limitation via transcriptional control of *rfaH* gene (Bittner et al., 2002). Additionally, in *Bradyrhizobium japonicum*, a nitrogen-fixing bacterium, deletion of σ54 (*rpoN1, rpoN2*, and both) led to significant decrease in viability during desiccation stress (Cytryn et al., 2007). WecD, TDP-fucosamine acetyltransferase, is required in the final step for the synthesis of 4-acetamido-4,6-dideoxy-d-galactose, a sugar unit of polysaccharide (O antigen) which is composed of repeating unit of trisaccharide (Hung et al., 2006).

Dam, DNA adenine methylase, plays important role in DNA replication, DNA mismatch repair and SOS response (a genome-wide response to DNA damage where cell cycle is arrested and DNA repair and mutagenesis is active; Stephenson and Brown, 2016). Dam plays a protective role during oxidative stress in *S*. Typhimurium (Chatti et al., 2012). XerCD are site-specific tyrosine recombinase genes that resolve chromosome dimer (and is lethal if not resolved) at a *dif* site (Dörr et al., 2009). *xerC* mutant of *Staphylococcus aureus* demonstrated limited biofilm formation and attenuated virulence in murine bacteremia model (Atwood et al., 2016).

### Other desiccation survival genes

Hfq, an RNA chaperone protein, has a diverse role in bacterial physiology including growth-dependent metabolism, stress resistance, virulence and drug resistance through post-transcriptional control of gene expression. The most prominent role of Hfq protein in bacteria is in facilitating the interactions between non-coding sRNAs and their cognitive target mRNA molecules (De Lay et al., 2013). Although no sRNA genes implicated in desiccation survival has been reported, the importance of *hfq* gene in desiccation survival of *S*. Typhimurium may suggest the involvement of unknown sRNAs in the process. The 6 intergenic regions identified in this study to be required for desiccation survival may support this hypothesis (see the next section). In *Francisella novicida*, Hfq protein has an important role in resistance to stresses such as, osmotic change, low pH, heat shock and oxidative stress. *Salmonella* Hfq protein positively regulates virulence by targeting *hilD* mRNA that affects secretion of type III secretion system (T3SS) encoded by *Salmonella* pathogenicity island 1 (SPI-1) (Shakhnovich et al., 2009; Yang et al., 2015). SPI-2 genes (*ssaHIJ*) encoding T3SS were also required for desiccation survival of *Salmonella*. TolB, a translocation periplasmic protein, is involved in maintaining the integrity of outer membrane via Tol/Pal system in *E. coli* (Walburger et al., 2002).

MiaA, a tRNA delta (2)-isopentenyl pyrophosphate transferase gene, is required for the efficient translation of the *rpoS* (σ^S^) mRNA. σ^S^ factor is necessary for the stationary phase/general stress response and required during nutrient starvation and presence of toxic metabolite in *E. coli* (Thompson and Gottesman, 2014). However, *rpoS* was not identified as desiccation survival gene in this study. Pnp, a polynucleotide phosphorylase/polyadenylase, provides protection against lactic acid exposure in *S*. Typhimurium. Moreover, *pnp* mutant in *E. coli* has a decrease in RpoS-regulated transcripts (Bearson et al., 2006). Null mutations of *wecE* gene (TDP-4-oxo-6-deoxy-D-glucose transaminase) in *E. coli* responsible for the synthesis of enterobacterial common antigen (ECA), a glycolipid found in the outer leaflet of the outer membrane in all species of family *Enterobacteriaceae*, confers sensitivity to bile (Danese et al., 1998). PagO, an integral inner membrane protein, is activated by *phoPQ* regulon. *Salmonella* PhoPQ is a two-component regulatory system that provides protection against host cationic antimicrobial peptides and intracellular survival within acidic phagosomes by regulating outer membrane (OM) acidic glycerophospholipids with lipid A structure (Dalebroux et al., 2014). *Salmonella* lacking *tpiA* gene, encoding a glycolytic enzyme triosephosphate isomerase that plays a key role in the central carbon metabolism, has an altered morphology with an elongated shape as compared to the wild type and is required for full *in vivo* fitness (Paterson et al., 2009).

Additionally, genes encoding putative proteins required for desiccation survival included inner membrane protein (STM14_3329, STM14_2258, and STM14_3328); glycosyl transferase (STM14_0839 and STM14_0845); STM14_1490 (*envF*, putative envelope protein); STM14_1486 (putative cytoplasmic protein); STM14_2014 (putative thiol peroxidase); and STM14_0838 (putative UDP-galactopyranose mutase). Similarly, *S*. Typhimurium desiccation tolerance genes encoding hypothetical proteins were STM14_0872 (*ybgF*), STM14_1487, STM14_2015, STM14_3164 (*gogB*), STM14_3165, and STM14_4907 (*yiiQ*). YbgF (*ybgC*-*tolQRAB*-*pal*-*ybgF* operon) is involved in maintenance of cell envelope integrity. GogB, a phage-encoded effector protein, is an anti-inflammatory effector, which regulates inflammation-enhanced colonization and limits tissue damage during *Salmonella* infection (Pilar et al., 2012).

### Desiccation resistance *Salmonella* intergenic regions

We identified six entirely essential intergenic regions of *S*. Typhimurium required for survival in desiccation stress on petri plate (Table 3). To determine if any of these intergenic regions encode non-coding sRNA, genomic DNA sequence was extracted for these intergenic regions and blasted for the presence of small RNA (sRNA) against sRNATarBase 2.0, a database of bacterial sRNA targets verified by experiment (Cao et al., 2010). However, we could not find hit for any known sRNA. There might be novel genetic elements in these intergenic regions of *Salmonella* genome yet to be explored.

**Table 3:** The intergenic regions of *S*. Typhimurium 14028S required for desiccation survival.

**Intergenic region**	**Start**	**End**	**Length (bp)**	**Essentiality**
IG_STM14_3329	2,923,580	2,923,838	259	2
IG_STM14_3165	2,782,023	2,782,225	203	2
IG_STM14_3164	2,780,125	2,780,528	404	2
IG_STM14_2257	1,971,827	1,971,958	132	2
IG_STM14_1490	1,337,373	1,338,163	791	2
IG_STM14_0255	253,521	253,756	236	2

*2- Entirely essential as classified by Con-ARTIST pipeline*.

Furthermore, we searched for the presence of coding region in the desiccation resistance intergenic region using GeneMark (http://www.ncbi.nlm.nih.gov/genomes/MICROBES/genemark.cgi). There was no coding sequence in the 5 intergenic regions. Strikingly, IG_STM14_1490 had a coding sequence with start at 1337639 bp and end at 1337842 bp of 204 bp. The result corroborates with PATRIC (Pathosystems Resource Integration Center, www.patricbrc.org) annotation that contains a hypothetical protein (fig|588858.6.peg.1457) on the negative strand. Moreover, we looked for the promoter regions in the desiccation resistance intergenic regions using Pepper (http://genome2d.molgenrug.nl/index.php/prokaryote-promoters). Interestingly, only IG_STM14_3165 had the predicted promoter.

### Phenotypic evaluation of single gene knockout mutants

**Figure 2:**
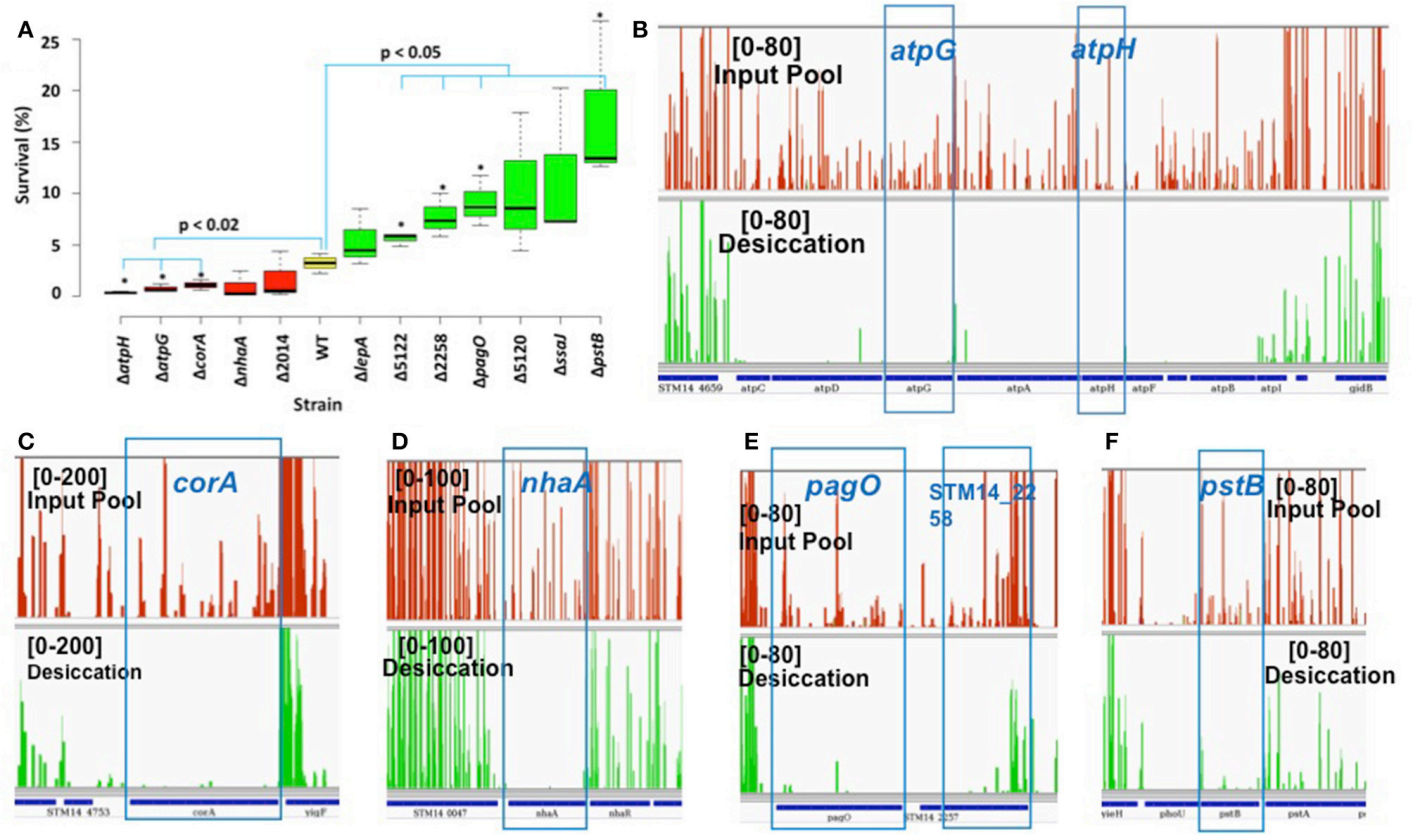
Phenotypic study of null mutants. **(A)** Box plot displays survival (%) of WT (*S*. Typhimurium, yellow) and mutants (red and green). Red and green color boxplot has mean survival (%) lower and greater than WT, respectively. Box represents first and third quartile, line inside the box is median and whisker shows minimum and maximum. Strain marked with an asterisk (^*^) have significantly different survival than the wild type. (Δ2014–ΔSTM14_2014, Δ5122–ΔSTM14_5122, and Δ5120–ΔSTM14_5120). **(B–F)** Presentation of Tn5 read coverage in input pool (red) and desiccation (green) produced using Integrative Genomics Viewer (IGV; Thorvaldsdottir et al., 2013). Numbers in the square is read coverage.

We performed phenotypic evaluation of 12 single knockout mutants to validate the functional roles of the genes in desiccation survival. Six *S*. Typhimurium knockout mutants were entirely essential (Δ*nahA*, Δ*atpG*, Δ*atpH*, Δ*ssaj*, Δ*lepA*, and Δ*pagO*) and six were domain essential (Δ*corA*, Δ*pstB*, ΔSTM14_2014, ΔSTM14_5120, ΔSTM14_2258, and ΔSTM14_5122). Tn-seq analysis showed the fold change in read numbers [log_2_(OP/IP)] of mutant strains varied from −7.98 to −0.6 with the difference of unique insertion count (DUIC; unique insertions of OP—unique insertions of IP) ranging from −86 to −10 (Table 2) calculated using Tn-Seq Explorer (Solaimanpour et al., 2015). Unique insertion count is the number of genomic loci disrupted by Tn5 insertion. Mutant survival (%) was calculated as described in Materials and Methods. The result showed that only five of the mutants demonstrated reduced survival as compared to the wild type and seven strains had higher survival rate than the wild type (Figure 2A). Among the five mutants with reduced desiccation survival, only three mutants (Δ*atpH*, Δ*atpG*, and Δ*corA*) showed statistically significant reduction in survival as compared to the wild type (*p* < 0.02, unpaired *t*-test). To surprise, four mutants had significantly increased survival compared to the wild type (ΔSTM14_5122, ΔSTM14_2258, Δ*pagO*, and Δ*pstB*) contrary to the results of Tn-seq analysis (Figure 2A). To understand the discrepancy between Tn-seq result and phenotypic data, we inspected Tn5 insertion profiles in IP and OP for these genes. The profiles show significantly reduced read numbers after the selection for each identified gene as shown in Figures 2B–F and Figures S1A–E, corroborating well with the genes identified by the analysis of Tn-seq data. Spearman correlation analysis also indicated that there was a significant correlation between survival (%) and log_2_FC (*R*^2^ = 0.62, *p* = 0.0307) as shown in Figure S2.

However, the result of the phenotypic study did not well substantiate the result of Tn-seq analysis for all mutants tested. We speculate that the disagreement is partially due to the differences in the assay conditions for the library selection and phenotypic assay for single mutants. They differ in terms of the context of experimental vessel (petri plate vs. 96 well plate) and cells (library vs. single mutant), drying method, duration of desiccation stress etc. During the process of optimizing the condition for phenotypic assay, we found that the survival rate of the wild type cells fluctuates greatly depending on the parameters used in the assays. Also, if the phenotype is influenced by the factors secreted into media, the phenotypic outcome of a mutant can be different depending on whether it exists in the context of a mutant library or the pure culture of the same mutant cells. Therefore, we expect that the use of further optimized assay condition may provide the results more consistent with the result of Tn-seq analysis for all mutants tested.

### Comparative study

We have searched for *Salmonella* genes in literature, which have been associated with desiccation resistance. Major genes involved in desiccation resistance were K^+^ transport channel *kdpFABC* transporter, isocitrate-lyase *aceA*, lipid A biosynthesis palmitoleoyl-acyltransferase *ddg*, iron-sulfur cluster scaffolding protein *nifU*, global regulator *fnr*, alternative sigma factor *rpoE* (Gruzdev et al., 2012b), specialized sigma factor *rpoS* (Finn et al., 2013a), osmoprotectant transporters (*proUP* and *osmU*) (Finn et al., 2013b), and trehalose biosynthesis genes (*ostAB*) (Li et al., 2012). All of these genes were disrupted by Tn5 in both IP and OP (Table S3), making them non-essential for survival during desiccation in our experimental setting. Furthermore, these mutants were not sensitive to desiccation stress in our study (Figure S3). We speculate that the discrepancy may be due to the differences in multiple factors, including the genetic backgrounds, the experimental settings (stainless steel surface, sterile filter paper or plastic ware), variable desiccation period (couple of hours to weeks), genomic techniques (transcriptome vs. Tn-seq), and/or sensitivity of Con-ARTIST pipeline to identify CEGs. Interestingly, trehalose-negative strains of *Cronobacter* spp. was shown to survive dry stress as well as the wild type strains, suggesting that the factors for desiccation survival could vary in different genetic backgrounds (Breeuwer, 2014).

**Figure 3:**
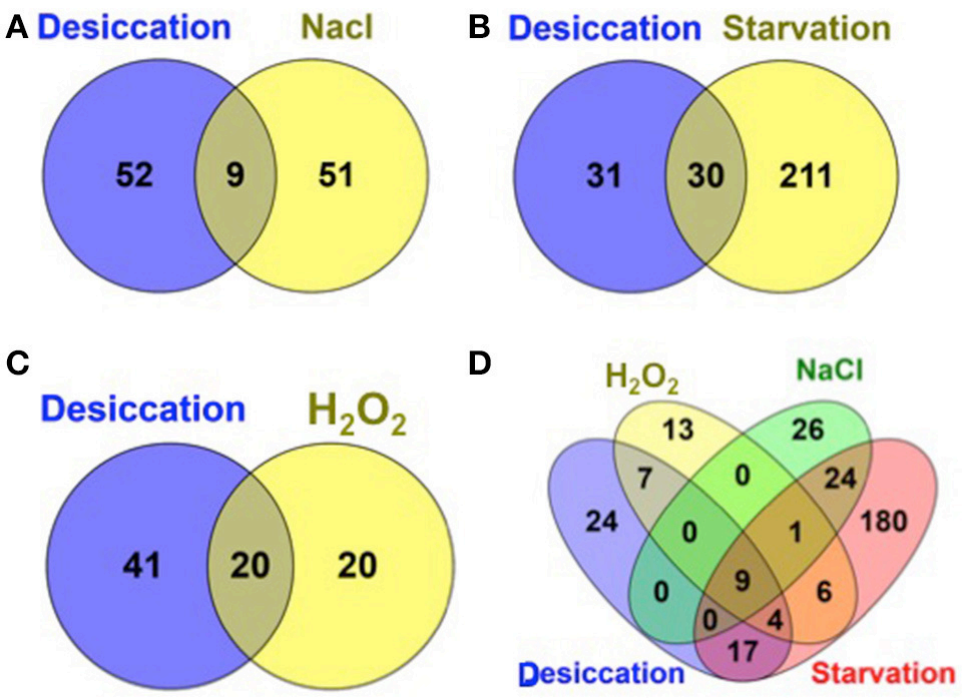
Venn diagram showing a comparison of *S*. Typhimurium desiccation resistance genes with other environmental stress resistance genes. Desiccation resistance genes compared with previously identified resistance genes (Mandal, 2016) during: **(A)** osmotic stress (3% NaCl in LB medium); **(B)** Starvation (starved for 12 days in PBS); **(C)** oxidative stress (1 mm hydrogen peroxide (H_2_O_2_) in LB medium); and **(D)** all the four stressors.

Additionally, we compared the desiccation resistance genes with those genes required for resistance against related environmental stresses such as, starvation, osmotic, and oxidative stress encountered during the infection cycle by *S*. Typhimurium from our recent study (Mandal, 2016) as shown in Figure 3. To note, the same IP used in this study was used for all screening against these stress conditions. Interestingly, we found a more than 50% of desiccation resistance genes were shared by the genes for starvation survival (*Salmonella* starved for 12 days) and more than 30% of desiccation resistance genes were shared by the genes required for resistance to hydrogen peroxide (H_2_O_2_, 1mM) stress. Hence, this may indicate that *S*. Typhimurium experience starvation as well as oxidative stress during desiccation. Additionally, only ATP synthase genes (9 subunit proteins) were shared between desiccation and osmotic stress (3% NaCl) that were also required for fitness during starvation and hydrogen peroxide insult (Figure 3). Thus, osmotic stress imposed by 3% NaCl is distinct from the osmotic stress incurred by *Salmonella* during desiccation stress.

## Conclusion

For the first time to our knowledge, we performed a genome-wide screening of a transposon mutant library to identify desiccation survival genes of *S*. Typhimurium. The precision and accuracy for the identification of CEGs depend on the complexity of the input library, experimental design, and downstream bioinformatics analysis. The outcome of Tn-seq data analysis depends on several factors like library normalization (bottleneck, positional read bias, differences in sequencing depth and the stochastic difference in library complexity), annotation dependent analysis and annotation-independent analysis (Chao et al., 2016). In this study, we used Con-ARTIST pipeline that enables the characterization of transposon mutant with annotation-independent approach for discovery of genetic elements at a sub-genic level. We identified 61 protein coding genes and six intergenic regions required for the survival of *S*. Typhimurium during a desiccation stress. The important resistance genes to survive the desiccation stress by *S*. Typhimurium were related to energy production and conversion required to maintain basal metabolism; cell wall/membrane/envelope biogenesis required for production of extracellular polysaccharide; post-translational modification, protein turnover, and chaperones; inorganic ion transport and metabolism for transport of magnesium, nickel, cobalt, sodium, iron and phosphate; replication, recombination and repair to overcome DNA damage; intracellular trafficking, secretion, and vesicular transport; translation, ribosomal structure and biogenesis and transcription. More than 20% of were either putative or hypothetical genes that helped assign novel functions to previously unknown genes. Few genes related to amino acid, nucleotide and carbohydrate transport and metabolism were also required to survive a desiccation stress encountered by *Salmonella*. Thus, our study was able to provide novel insights into the underlying mechanisms of desiccation survival of *Salmonella*. We expect that our findings can be further exploited to develop effective control strategies to control the *Salmonella* contamination from low water activity foods and food processing facilities.

## Author contributions

YMK: Conceived the study; RKM: performed experiment, analyzed the data, and drafted the manuscript; YMK: revised the manuscript.

### Conflict of interest statement

The authors declare that the research was conducted in the absence of any commercial or financial relationships that could be construed as a potential conflict of interest.
