# Supplementary material for: Global Screening of Salmonella enterica Serovar Typhimurium Genes for Desiccation Survival
Source: Front Microbiol. 2017 Sep 8;8:1723. doi: 10.3389/fmicb.2017.01723 (PMC5596212; doi:10.3389/fmicb.2017.01723)
Supplement: Supplementary file 1 [file DataSheet1.PDF]

Supplementary Material

Global screening of *Salmonella enterica* serovar Typhimurium genes for desiccation survival

Rabindra K. Mandal[1,3*] and Young Min Kwon[1, 2]

[1]Department of Poultry Science, University of Arkansas, Fayetteville, AR, 72701

[2]Cell and Molecular Biology Program, University of Arkansas, Fayetteville, AR, 72701

[3]*Corresponding author present address:

Department of Microbiology and Immunology

Clinical Translational Research Building

University of Louisville

Louisville, KY 40202

Phone: (479) 575-4935

Email: rabindra.mandal@louisville.edu

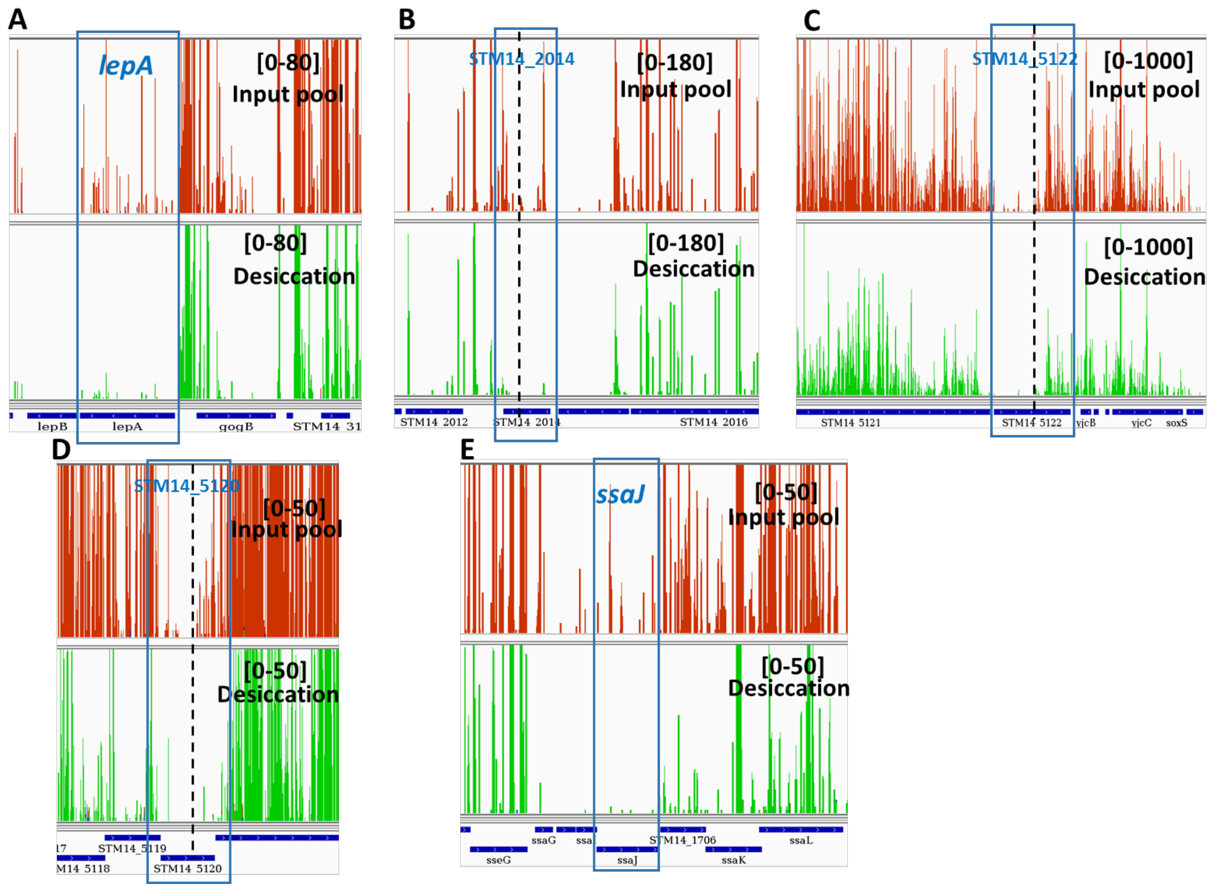

Figure S1: Read coverage in input pool (red) and desiccation in genes of interest. Graph is produced using Integrative Genomics Viewer (IGV). Numbers in square shows read coverage. Genes with dotted line inside blue box was domain essential.

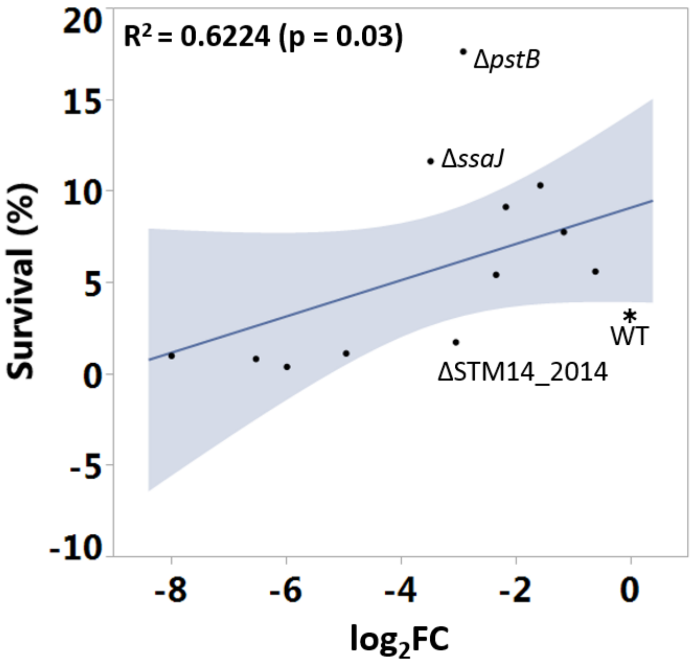

Figure S2: Spearman correlation between survival (%) of null mutants of this study and $\log_2$ fold change ($\log_2$FC). $\log_2$FC is calculated for each gene after normalization of input pool read count based on desiccation read count. More –ve $\log_2$FC of a gene implies more reduced fitness and is lower survival (%) during desiccation. Y-axis: Average survival (%) of 12 mutants and X-axis: $\log_2$FC of read distribution in central 80% of gene based on Tn-seq analysis. Line of fit shows linear regression with confidence intervals. * is survival (%) of wild type (WT) with hypothetical $\log_2$FC of zero (There would be no change in the read count for WT after desiccation).

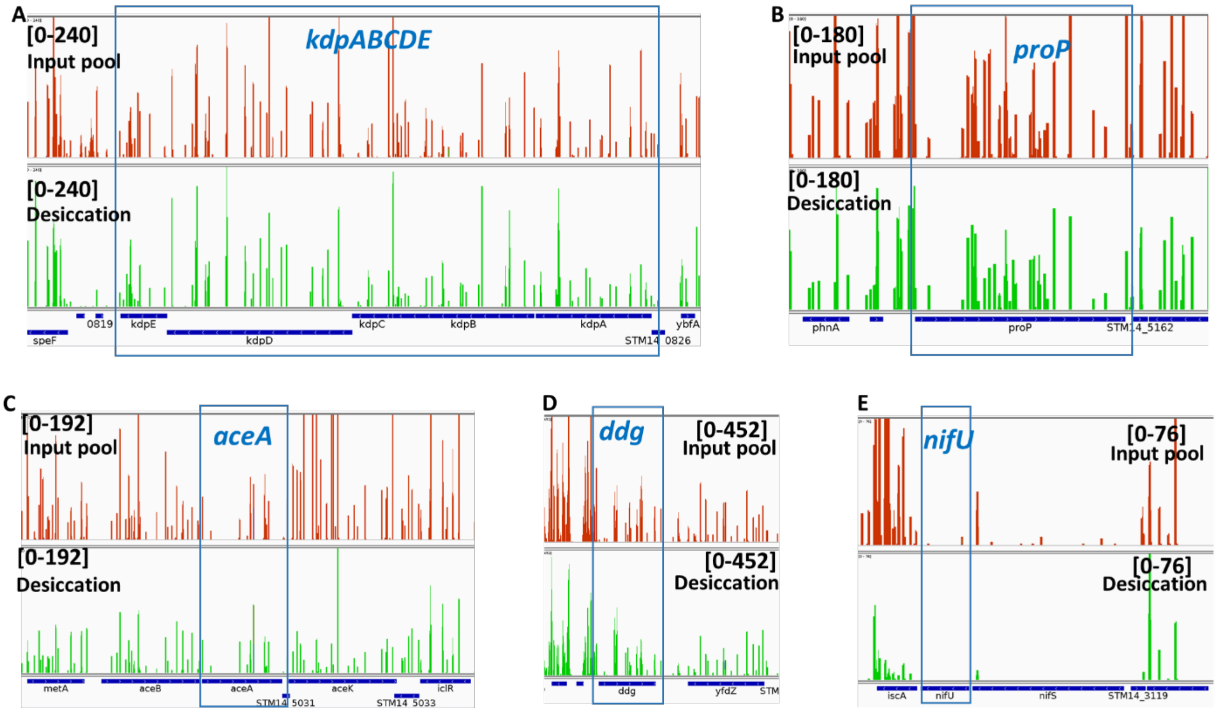

Figure S3: Tn5 insertion read coverage in the previously identified *S.* Typhimurium desiccation resistance genes. Graphs were generated using IGV. Tn5 insertion in K+ transport channel *kdpABCDE* transporter (A), isocitrate-lyase *aceA* (B), lipid A biosynthesis palmitoleoyl-acyltransferase *ddg* (C), iron-sulfur cluster scaffolding protein *nifU* (D) and osmoprotectant transporters *proP* (E).

Table S1: Oligonucleotide used for Tn-seq PCR

Tn-seq PCR	Oligonucleotide	Sequence	Barcode
Linear Extension	Ez-Tn5 primer3	5'-GATCCTCTAGAGTCGACCTGCAGGCATGCA-3'	
Exponential PCR	IR2-IS-B7	5'-AATGATACGGCGACCACCGAGATCTACACTCTT TCCCTACACGACGCTCTTCCGATCTNNNNAGXXXXXX TCAGGGTTGAGATGTGTATAAGAGACAG-3'	CAGATC
	IR2-IS-B14	5'-AATGATACGGCGACCACCGAGATCTACACTCTTT CCCTACACGACGCTCTTCCGATCTNNNNAGXXXXXX TCAGGGTTGAGATGTGTATAAGAGACAG-3'	CGTTGT
	HTM-Primer	5'-CAAGCAGAAGACGGCATACGAGCTCTTCCGATCT GGGGGGGGGGGGGGG-3'	

NNNN: random sequence for efficient cluster analysis. XXXXXX: 6nt sample barcode sequences. For exponential PCR, IR2-IS-B7 and HTM-primer were used for input pool and IR2-IS-B14 and HTM-primer was used for desiccation. Few wild characters (.C.G.G.T.A.A.G.G.A.A.GAGACAG in Perl script, where "." is any character) were used in mosaic end reads for identification of the reads containing Tn5-junction sequences.

Supplementary Protocol 1: DNA library preparation protocol for transposon sequencing (Tn-seq) using regular primer

This protocol for DNA library preparation of transposon mutant library is the improved version of previously developed methodology in our laboratory (1). This approach is based on addition of poly deoxycytosine (C) tails to 3' end of either single or double-stranded DNA (2). Addition of C tail is controlled effectively by the mixture of deoxycytidine triphosphate (dCTP) and dideoxy CTP (ddCTP). A single-primer is used for the linear extension of transposon junction sequences with a transposon-specific primer. The linear extension product is purified and subsequently C-tail is attached to it. Next, the transposon junction sequence is easily amplified by transposon specific primer and poly G primer (3). This improved version uses only one polymearase and minimizes the use of oligonucleotide and PCR amplification. The simplified diagram for improved version is shown in Figure S4. DNA from 300 bp – 500 bp is extracted from 1.5% agarose gel as shown in Figure S5.

MATERIALS

DNA extracted from Tn5 mutant library
Wild type DNA (Control DNA)
QIAamp DNA Mini Kit (Qiagen, Valencia, CA, USA)
Qubit 2.0 Fluorometer (Life Technologies, Carlsbad, CA)
Oligonucleotide (Table S1)
Taq DNA polymerase (New England Biolabs, Ipswich, MA, USA)
Thermopol buffer
2.5 mM dNTP mix
Nuclease-free water
MinElute PCR purification kit (Qiagen, Valencia, CA, USA)

Terminal Transferase (TdT, New England Biolabs, Ipswich, MA, USA)

TdT Reaction Buffer (10X)

CoCl2 (2.5 mM) (TdT, New England Biolabs, Ipswich, MA, USA)

dCTP (100 mM) (Promega, Madison, WI, USA)

ddCTP (10 mM) (Promega Madison, WI, USA)

1.5% Agarose gel

QIAquick Gel Extraction Kit (Qiagen, Valencia, CA, USA)

Thermocycler (PCR machine)

STEPS

Step1: Linear extension PCR

1. Reaction mixture

ddH_2O	40 µl
Thermopol Buffer (10X)	5 µl
dNTPs (2.5 mM each)	1 µl
EZ-Tn5 primer 3 (20 µM) (T_m=65.6ºC)	1 µl
Genomic DNA of Tn library	2 µl
Taq DNA polymerase (NEB)	1 µl
Total	50 µl

2. PCR cycle

	95°C	2 min (manual hot-start)
50 cycles	[95°C	30 sec]
	[62°C	45 sec]
	[72°C	10 sec]
	4°C	hold

3. Purify the linear extension PCR products using Qiagen MinElute PCR-purification kit. Elute DNA in 10 µl EB buffer and store at -20°C.

Step 2: C-tailing reaction

1. Preparation of dNTP working stock

   Dilute 100 mM dCTP to 10 mM dCTP with ddH_2O (nuclease-free)
   Dilute 10 mM ddCTPto 1 mM ddCTP with ddH_2O (nuclease-free)

2. Reaction mixture

DNA (linear extension products)	10.0 µl
TdT Buffer (10X)	2.0 µl
2.5 mM $CoCl_2$	2.0 µl
10 mM dCTP	2.4 µl
1 mM ddCTP	1.0 µl
ddH_2O	1.6 µl
Terminal transferase	1.0 µl
Total	20.0 µl

3. Incubate the reaction tube at 37°C for 1 hr.
4. Incubate the reaction tube at 75°C for 20 min for heat inactivation of TdT.
5. Purify the C-tailed products using Qiagen MinElute PCR-purification kit. Elute DNA in 10 µl EB buffer and store at -20°C.

Step 3: PCR to amplify Tn-flanking sequences

1. Reaction mixture

ddH_2O	35 µl
Thermopol Buffer (10X)	5 µl
dNTPs (2.5 mM each)	4 µl
IR2-IS-BC primer (10 µM)	2 µl
HTM primer (20 µM)	1 µl
C-tailed DNA	2 µl
Taq DNA polymerase (NEB)	1 µl
Total	50 µl

Note- IR2-IS-BC primer is barcoded primer used during exponential PCR.

4. PCR cycle

	95°C	2 min (manual hot-start)
36 cycles	[95°C	30 sec]
	[58°C	45 sec]
	[72°C	20 sec]
	72°C	10 min
	4°C	hold

Step 4: Gel-purification of PCR products

1. Mix the sample with loading buffer and heat at 65ºC for 15 min.
2. Run 10 µl/sample on 1.5% agarose gel.
3. Cut 300-500bp bands and gel-purify DNA fragments.

References (Supplementary File 1):

1. **Dawoud TM, Jiang T, Mandal RK, Ricke SC, Kwon YM.** 2014. Improving the efficiency of transposon mutagenesis in Salmonella enteritidis by overcoming host-restriction barriers. Mol Biotechnol **56:**1004-1010.

2. **Lazinski DW, Camilli A.** 2013. Homopolymer tail-mediated ligation PCR: a streamlined and highly efficient method for DNA cloning and library construction. BioTechniques **54:**25.

3. **Kwon YM, Ricke SC, Mandal RK.** 2016. Transposon sequencing: methods and expanding applications. Appl Microbiol Biotechnol **100:**31-43.

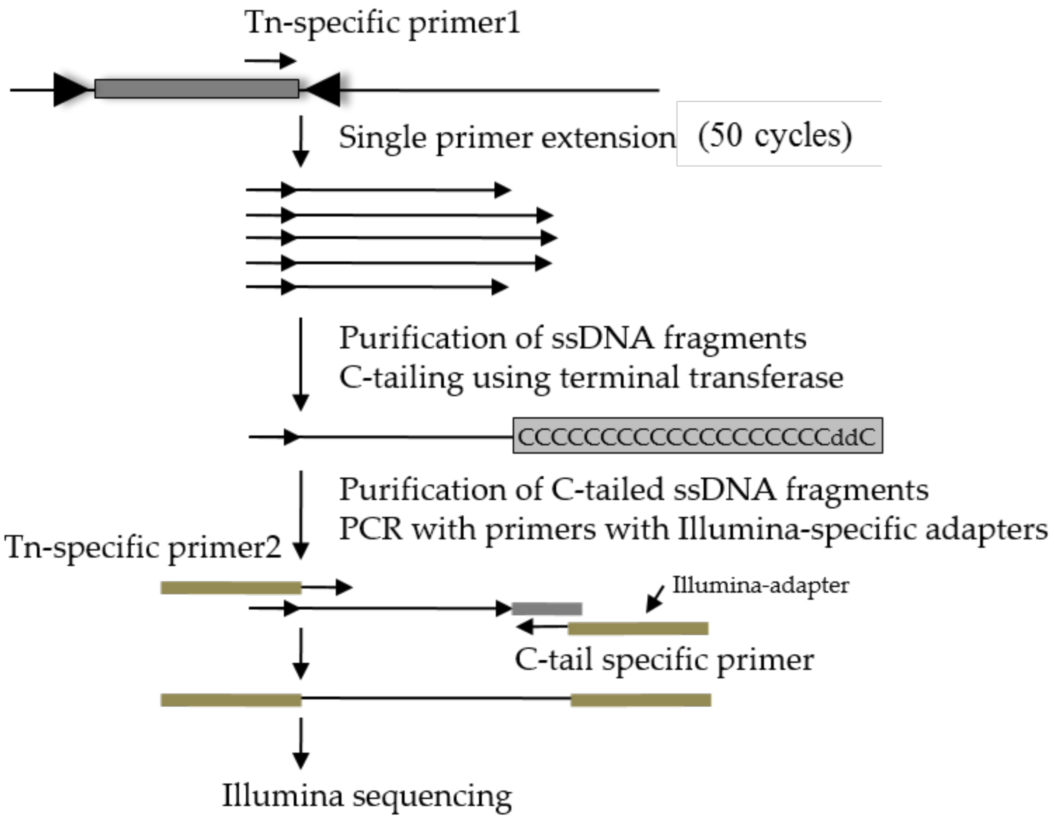

Figure S4: Schematic diagram of DNA library preparation of transposon library for Illumina sequencing. Tn-specific primer 1 (Ez-Tn5 primer3, Table S1) is used for linear extension and Tn-specific primer 2 (library specific barcoded primer according) is used for exponential PCR in conjunction with C-tail specific primer (HTM-Primer, Table S1).

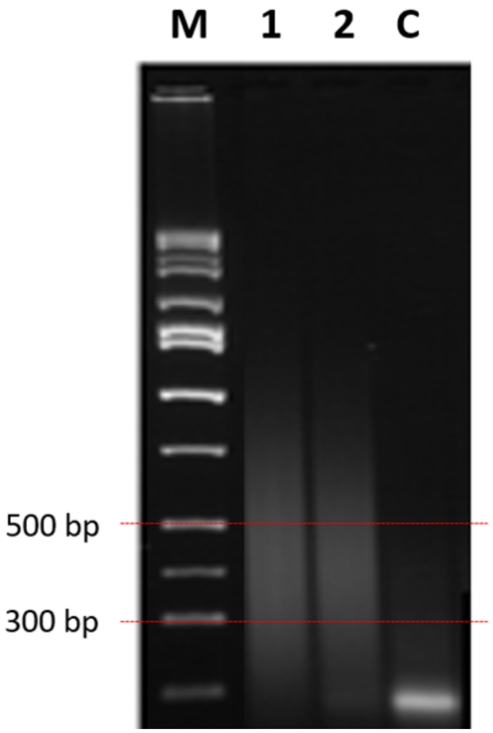

Figure S5: Agarose gel (1.5%) run of DNA library after exponential PCR (Step 4). DNA from 300 bp – 500 bp is extracted from gel using QIAquick Gel Extraction Kit. M: Hi-Lo Marker, 1: Input pool, 2: Desiccation, C: Control (Wild Type DNA).
